# Associations and predictive performance of 11 anthropometric measures with incident type 2 diabetes: A prospective cohort study from the UK Biobank

**DOI:** 10.1002/oby.23849

**Published:** 2023-09-19

**Authors:** Jirapitcha Boonpor, Solange Parra‐Soto, Atefeh Talebi, Ziyi Zhou, Fernanda Carrasco‐Marin, Fanny Petermann‐Rocha, Paul Welsh, Jill P. Pell, Naveed Sattar, Jason M. R. Gill, Stuart R. Gray, Carlos Celis‐Morales, Frederick K. Ho

**Affiliations:** ^1^ School of Cardiovascular and Metabolic Health University of Glasgow Glasgow UK; ^2^ Faculty of Public Health Kasetsart University Sakon Nakhon Thailand; ^3^ Department of Nutrition and Public Health Universidad del Bío‐Bío Chillan Chile; ^4^ School of Health and Wellbeing University of Glasgow Glasgow UK; ^5^ Centre for Healthy Living Universidad de Concepción Concepción Chile; ^6^ Center for Biomedical Research, Faculty of Medicine Universidad Diego Portales Santiago Chile; ^7^ Research Group on Education, Physical Activity and Health (GEEAFyS) University Católica del Maule Talca Chile

## Abstract

**Objective:**

The study aim was to investigate associations of 11 anthropometric measures with incident type 2 diabetes and compare their predictive performance.

**Methods:**

This prospective cohort study included 161,127 White European UK Biobank participants who were free of diabetes at baseline. Anthropometric measures included height, weight, BMI, A Body Shape Index, waist circumference, waist to hip ratio, waist to height ratio (WHtR), hip circumference, visceral adiposity index, hip index, and anthropometric risk index. The associations were examined using Cox proportional hazard models. The differences in C‐index were used to compare predictive performance between BMI and other anthropometric measures.

**Results:**

The median follow‐up was 10.0 (interquartile range: 9.3–10.8) years, during which 6315 participants developed type 2 diabetes. All markers except height and hip index were positively associated with incident type 2 diabetes. The strongest associations were found for WHtR (hazard ratio per 1‐SD increment: 2.27 [95% CI 2.19–2.35] in women; 1.96 [95% CI 1.90–2.01] in men). Compared with BMI, WHtR and anthropometric risk index had significantly better type 2 diabetes risk discrimination.

**Conclusions:**

Although most adiposity markers were associated with type 2 diabetes, the magnitude of the associations differed. WHtR had the strongest associations and predictive ability for type 2 diabetes and thus could be a more suitable marker for clinical use.

**Study Importance**

**What is already known?**

- Anthropometric measurements have been used to predict the risk of type 2 diabetes. However, the predictive performance was found to be inconsistent.

**What does this study add?**

- Most anthropometric measurements were positively associated with incident type 2 diabetes. However, waist to height ratio (WHtR) and anthropometric risk index had a significantly better predictive performance for type 2 diabetes risk than BMI.
- Being shorter and having smaller hips were associated with a higher risk of type 2 diabetes.

**How might these results change the direction of research or the focus of clinical practice?**

- More complex markers did not outperform simple, conventional measures such as BMI and WHtR.
- WHtR had the strongest associations and predictive ability for type 2 diabetes and thus could be a more suitable marker for clinical use.

## INTRODUCTION

Type 2 diabetes is a major public health challenge linked to a higher risk of noncommunicable diseases, such as cardiovascular disease, chronic kidney disease, and premature death [1]. It is well known that obesity is strongly associated with developing type 2 diabetes [2, 3, 4]. Adiposity quantified by body mass index (BMI) and waist circumference (WC) is a strong predictor for type 2 diabetes development [2, 5, 6]. However, other anthropometric markers, such as waist to height ratio (WHtR) [6], have been shown to be better predictors for obesity‐related comorbidities than BMI [7]. Moreover, various new anthropometric indices for adiposity have been developed, for example, visceral adiposity index (VAI), A Body Shape Index (ABSI), hip index (HI), and anthropometric risk index (ARI) [8, 9, 10, 11]. These markers have been associated with cardiometabolic risk and mortality [9, 10], but as with WHtR, their associations with type 2 diabetes still require further study [12].

A recent systematic review and meta‐analysis examined the associations between various anthropometric markers and type 2 diabetes risk [12]. However, the number of studies included was small, and they were subject to between‐study confounding in their dose–response analysis [12]. That study also did not examine the prediction utility of these markers and it was not separated by sex.

Therefore, our study aimed to investigate the dose–response associations of anthropometric markers (body height and weight, ABSI, BMI, WC, waist to hip ratio [WHR], WHtR, hip circumference [HC], VAI, HI, and ARI) with incident type 2 diabetes in the UK Biobank, a large prospective cohort study. This study also explored whether these associations differed by sex and compared the markers' predictive performance.

## METHODS

### Data source

The UK Biobank study recruited more than 502,000 participants between 2006 and 2010 (5.5% response rate, men and women aged 37–73 years) from the general population [13]. Participants attended 1 of 22 assessment centers across England, Wales, and Scotland [14, 15]. Participants completed electronic consent, touch screen questionnaires, and physical measurements at the assessment centers, including anthropometric measurements. The current study included 161,127 White European participants who had data available for incident type 2 diabetes, anthropometric markers, and covariates. Participants were excluded if they had prevalent type 1, type 2, or undiagnosed diabetes (hemoglobin A_1c_ ≥48 mmol/mol) at the baseline assessment as well as if they had a non‐White European background due to ethnic differences in adiposity. In addition, participants with missing data on exposures and covariates or those who developed type 2 diabetes in the first 2 years were excluded from the present study (Supporting Information Figure S1).

The UK Biobank study was approved by the North West Multi‐Centre Research Ethics Committee (Ref. 11/NW/0382 on June 17, 2011), and all participants provided written informed consent to participate.

### Outcome

Incident type 2 diabetes was derived from linkage to primary care data in the UK Biobank. Records were extracted for 45% of the UK Biobank cohort (228,449 participants). The end of coverage (extract date) was September 2021. Detailed linkage procedures are available at http://biobank.ndph.ox.ac.uk/showcase/showcase/docs/primary_care_data.pdf. We defined incident type 2 diabetes as primary care diagnosis with *International Classification of Diseases, 10th Revision* (ICD‐10) code E11. The Read Codes used in the primary care data were converted into ICD‐10 codes using the UK Biobank's lookup table.

### Exposures

The exposures included 11 anthropometric adiposity‐related markers, that is, height, weight, ABSI, BMI, WC, WHR, WHtR, HC, VAI, HI, and ARI. The measurements were undertaken by trained staff using standardized protocols across the assessment centers at baseline. Height was measured to the nearest centimeter, using a Seca 202 stadiometer, and body weight to the nearest 0.1 kg, using a Tanita BC‐418 body composition analyzer. BMI was calculated as body weight in kilograms divided by height in meters squared and classified into the following categories: underweight (<18.5 kg/m^2^), normal weight (18.5 ≤ 25 kg/m^2^), overweight (25 ≤ 30 kg/m^2^), or obesity (≥30 kg/m^2^) [16]. The natural indent was used to measure WC (the umbilicus was used if the natural indent could not be observed) using a nonelastic Seca 200 tape. ABSI was calculated based on WC, BMI, and height, as shown in Supporting Information Table S1 [11]. HC was recorded at the widest part of the hips. WHR and WHtR are the ratio of WC to HC and the ratio of WC to height, respectively. HI was calculated from HC, weight, and height (Supporting Information Table S1) [17]. VAI was calculated based on BMI, WC, triglycerides, and high‐density lipoprotein cholesterol [10]. ARI was calculated by the sum of height, BMI, ABSI, and HI, as explained elsewhere [8].

### Covariates

Age was calculated from the date of birth and baseline assessment; sex was self‐reported. The deprivation index, an area‐based measure of socioeconomic status, was derived from the postal code of residence using the Townsend deprivation index [18]. Fruit and vegetable, red meat, and processed meat intakes were recorded using a touch screen questionnaire asking about the reported frequency of consumption. Alcohol intake was self‐reported and categorized as daily or almost daily, three to four times a week, once or twice a week, one to three times a month, special occasions only, and never. Smoking status was categorized into never, former, and current. Leisure screen time was self‐reported as discretionary screen time, TV viewing, and leisure PC screen time in hours per day. Sleep duration was categorized as short sleep (<7 h/d), normal sleep (7–9 h/d), and long sleep (>9 h/d) [19]. Type of physical activity was self‐reported in relation to five groups: walking for pleasure, other exercise (e.g., swimming, cycling), strenuous sports, light do‐it‐yourself (e.g., pruning, watering the lawn), and heavy do‐it‐yourself (e.g., weeding, lawn mowing, carpentry, digging). A family history of diabetes was self‐reported at baseline. Systolic blood pressure was derived from the mean of two readings recorded in the left arm using a standardized protocol. Additional details about these measurements can be found in the UK Biobank online protocol [20].

### Statistical analyses

Continuous variables are expressed as means with their respective standard deviations (mean [SD]), and categorical variables are presented as frequencies and percentages. The Pearson correlation coefficients were used to evaluate the correlations between variables. Cox proportional hazard models were used to investigate the associations of anthropometric markers, standardized by sex (expressed as 1 SD), with incident type 2 diabetes, with follow‐up as the timeline variable. Results are reported as hazard ratios (HRs) together with 95% confidence intervals (CIs), representing the ratio of hazards averaged across the follow‐up period [21]. The association analyses were conducted within a 2‐year landmark period and they excluded all participants with prevalent or undiagnosed diabetes at the baseline assessment or those with missing data on exposures and covariates to reduce reverse causation (Supporting Information Figure S1). Due to ethnic differences in adiposity, inclusion in the study was restricted to participants of a White European background.

The associations of anthropometric markers with incident type 2 diabetes were adjusted for covariates using two models with an increasing number of covariates. Model 1 (minimally adjusted model) was adjusted for sex, age, and deprivation index. Model 2 (lifestyle model) was adjusted for all variables in model 1 and additionally smoking, fruit and vegetable intake, red meat intake, processed meat intake, alcohol intake, type of physical activity, total sedentary time, and sleep duration. These were adjusted because they were likely to be confounders of the associations.

Women to men ratios of HRs were then estimated using Cox proportional hazard models with sex by anthropometric marker interaction terms. This term represents the statistical interaction between sex and the predictor and it can be interpreted as the ratio of HRs in females to that in males.

In the predictive analysis comparing type 2 diabetes risk discrimination between BMI and the remaining markers, we calculated Harrell's C index (the probability of concordance between observed and predicted responses) from a Cox model that included the markers and covariates (age, sex, systolic blood pressure, and family history of diabetes). These covariates, instead of the one in the association analysis, were chosen because they were commonly used in the clinical prediction model of type 2 diabetes, and in this analysis, we are interested in the predictive performance, which would not be affected by confounding. No 2‐year landmark analysis was used because reverse causation is not a concern in predictive analysis. BMI was a baseline model used to compare with models replacing BMI with other anthropometric markers. The C index differences between the models using BMI and other anthropometric parameters were calculated. The variance of the C indices was calculated using the formula previously described [22]. These were then used to calculate 95% CIs and *p* values using the normal approximation.

Nonlinear analyses were also conducted to investigate the associations of sex‐specific *z* scores of anthropometric markers with incident type 2 diabetes. Nonlinear associations were examined using penalized cubic splines fitted in Cox proportional hazard models. The penalized spline is a variation of the basis spline, which is not as sensitive to knot numbers and placements as restricted cubic splines [23]. The likelihood ratio tests were used to compare the models using splines and those assuming linearity.

Statistical analyses were performed using the statistical software Stata 17 (StataCorp LLC) and R 4.0.2 with the survival, compareC, psych, and corrplot packages; *p* < 0.05 was regarded as statistically significant.

## RESULTS

A total of 161,127 (55.0% women) participants with available data for incident type 2 diabetes, anthropometric adiposity‐related markers, and covariates were included in this study (Supporting Information Figure S1). After excluding the first 2 years, the median follow‐up period was 10.0 years (interquartile range: 9.3–10.8). Over the follow‐up, 6315 (3.9%) participants were diagnosed with incident type 2 diabetes (2638 women [1.6%] and 3677 men [2.3%]).

The overall cohort characteristics by BMI categories are shown in Table 1. In summary, the average age was 56.6 (SD 8.0) years. Participants classified as having obesity (22.0%) were older and more deprived than their counterparts with a lower BMI. The participants with obesity had a higher proportion of previous smokers, an alcohol intake of one to three times a month, and higher sedentary time. The cohort characteristics of women and men were similar. However, men had more daily alcohol drinking and higher BMI as presented in Supporting Information Table S2.

**TABLE 1:** Baseline characteristics by BMI categories

Variable	Underweight (*n* = 771, 0.5%)	Normal weight (*n* = 54,289, 33.7%)	Overweight (*n* = 70,566, 43.8%)	Obesity (*n* = 35,501, 22.0%)	Overall (*n* = 161,127)
Sex, *n* (%)					
Women	624 (80.9)	35,756 (65.9)	33,455 (47.4)	18,722 (52.7)	88,557 (55.0)
Men	147 (19.1)	18,533 (34.1)	37,111 (52.6)	16,779 (47.3)	72,570 (45.0)
Age (y), mean ± SD	55.5 ± 8.1	55.9 ± 8.1	57.1 ± 8.0	56.7 ± 7.8	56.6 ± 8.0
Townsend deprivation index, *n* (%)					
Lower deprivation	237 (30.7)	20,328 (37.4)	25,969 (36.8)	11,102 (31.3)	57,636 (35.8)
Middle deprivation	250 (32.4)	19,123 (35.2)	24,949 (35.4)	12,320 (34.7)	56,642 (35.2)
Higher deprivation	284 (36.8)	14,838 (27.3)	19,648 (27.8)	12,079 (34.0)	46,849 (29.1)
Smoking status, *n* (%)					
Never	465 (60.3)	32,469 (59.8)	38,140 (54.1)	18,496 (52.1)	89,570 (55.6)
Previous	154 (20.0)	16,266 (30.0)	25,904 (36.7)	13,910 (39.2)	56,234 (34.9)
Current	152 (19.7)	5554 (10.2)	6522 (9.2)	3095 (8.7)	15,323 (9.5)
Alcohol intake, *n* (%)					
Daily or almost daily	185 (24.0)	12,309 (22.7)	15,474 (21.9)	5897 (16.6)	33,865 (21.0)
3‐4 Times a week	148 (19.2)	14,111 (26.0)	18,209 (25.8)	7522 (21.2)	39,990 (24.8)
Once or twice a week	155 (20.1)	14,339 (26.4)	19,192 (27.2)	9941 (28.0)	43,627 (27.1)
1‐3 Times a month	86 (11.2)	5544 (10.2)	7510 (10.6)	4853 (13.7)	17,993 (11.2)
Special occasions only	99 (12.8)	4790 (8.8)	6315 (9.0)	4738 (13.4)	15,942 (9.9)
Never	98 (12.7)	3196 (5.9)	3866 (5.5)	2550 (7.2)	9710 (6.0)
Fruits and vegetables (portions/d), mean ± SD	4.2 ± 2.8	4.2 ± 2.3	4.1 ± 2.3	4.0 ± 2.3	4.1 ± 2.3
Red meat (portions/wk), mean ± SD	1.6 ± 1.4	1.9 ± 1.3	2.2 ± 1.4	2.3 ± 1.5	2.1 ± 1.4
Processed meat (portions/wk), mean ± SD	1.5 ± 1.2	1.7 ± 1.1	1.9 ± 1.0	2.0 ± 1.0	1.9 ± 1.0
Leisure screen time (h/d), mean ± SD	4.0 ± 1.9	4.5 ± 1.9	5.1 ± 2.1	5.6 ± 2.3	5.0 ± 2.2
Sleeping time (h/d), *n* (%)					
<7	550 (71.3)	42,252 (77.8)	53,445 (75.7)	25,173 (70.9)	121,420 (75.4)
7‐8	205 (26.6)	11,462 (21.1)	16,104 (22.8)	9620 (27.1)	37,391 (23.2)
>9	16 (2.1)	575 (1.1)	1017 (1.4)	708 (2.0)	2316 (1.4)
Type of PA, *n* (%)					
Walking for pleasure	621 (80.5)	43,705 (80.5)	54,799 (77.7)	25,313 (71.3)	124,438 (77.2)
Other exercises	76 (9.9)	6273 (11.6)	8913 (12.6)	5035 (14.2)	20,297 (12.6)
Strenuous sports	2 (0.3)	434 (0.8)	606 (0.9)	254 (0.7)	1296 (0.8)
Light DIY	56 (7.3)	2836 (5.2)	4394 (6.2)	3648 (10.3)	10,934 (6.8)
Heavy DIY	16 (2.1)	1041 (1.9)	1854 (2.6)	1251 (3.5)	4162 (2.6)
BMI (kg/m^2^), mean ± SD	17.7 ± 0.8	22.9 ± 1.5	27.3 ± 1.4	33.5 ± 3.5	27.1 ± 4.5
Height (cm), mean ± SD	166.8 ± 8.7	167.9 ± 8.9	169.4 ± 9.4	168.0 ± 9.4	168.6 ± 9.3
WC (cm), mean ± SD	66.1 ± 5.6	78.4 ± 8.0	90.6 ± 8.4	103.6 ± 10.4	89.2 ± 12.8
Weight (kg), mean ± SD	49.3 ± 5.8	64.7 ± 8.4	78.5 ± 9.6	94.9 ± 13.5	77.3 ± 15.2
ABSI, mean ± SD	0.08 ± 0.006	0.08 ± 0.005	0.08 ± 0.005	0.08 ± 0.005	0.08 ± 0.005
HC (cm), mean ± SD	86.8 ± 4.3	96.2 ± 4.8	103.1 ± 4.8	113.4 ± 8.5	103.0 ± 8.6
WHR, mean ± SD	0.8 ± 0.06	0.8 ± 0.07	0.9 ± 0.08	0.9 ± 0.09	0.9 ± 0.09
WHtR, mean ± SD	0.4 ± 0.03	0.5 ± 0.04	0.5 ± 0.04	0.6 ± 0.06	0.5 ± 0.07
ARI, mean ± SD	−2.8 ± 0.5	−1.6 ± 0.7	−0.1 ± 0.8	2.0 ± 0.9	−0.1 ± 1.6
HI women, mean ± SD	0.09 ± 0.005	0.07 ± 0.005	0.07 ± 0.004	0.06 ± 0.005	0.07 ± 0.008
HI men, mean ± SD	0.09 ± 0.004	0.08 ± 0.004	0.07 ± 0.003	0.07 ± 0.004	0.07 ± 0.006
VAI women, mean ± SD	1.1 ± 0.9	1.4 ± 1.0	2.1 ± 1.5	2.7 ± 1.8	1.9 ± 1.5
VAI men, mean ± SD	0.8 ± 0.6	1.5 ± 1.2	2.3 ± 1.6	3.0 ± 2.0	2.2 ± 1.7

*Note*: Data are presented as mean ± SD for continuous variables and as *n* (%) for categorical variables.

Abbreviations: ABSI, A Body Shape Index; ARI, anthropometric risk index; DIY, do‐it‐yourself; HC, hip circumference; HI, hip index; PA, physical activity; WC, waist circumference; WHR, waist to hip ratio; WHtR, waist to height ratio; VAI: visceral adiposity index.

A correlation matrix among the 11 anthropometric markers is shown in Supporting Information Figure S2. Overall, BMI showed a strong correlation with ARI, WHtR, HC, weight, and WC (*r* > 0.80). The weakest correlations were observed for BMI with HI, height, and ABSI (*r* < 0.20) (Supporting Information Figure S2).

The associations among the 11 anthropometric markers and incident type 2 diabetes are presented in Table 2. When the associations between the 11 anthropometric markers and incident type 2 diabetes were stratified by sex, women had a higher type 2 diabetes risk compared with men (sex interaction in Table 2). After adjusting for all covariates (Model 2), a 1‐SD increment for most markers was associated with a higher risk of type 2 diabetes. In women, a 1‐SD increment in WHtR was associated with a risk of type 2 diabetes that was 2.3 times higher (HR 2.27 [95% CI: 2.19–2.35]), followed by 2.0 times (HR 2.03 [95% CI: 1.96–2.10]) higher for WHR and 1.9 times (HR 1.93 [95% CI: 1.88–1.99]) higher for BMI. In men, the risk of type 2 diabetes with a 1‐SD increment in WHtR was greater by 96% (HR 1.96 [95% CI: 1.90–2.01]), followed by 85% (HR 1.85 [95% CI: 1.80–1.90]) for BMI and 77% (HR 1.77 [95% CI: 1.73–1.82]) for weight (Table 2). However, a 1‐SD increment of height and HI was associated with a lower risk of type 2 diabetes for both women and men (Table 2). The associations for all participants are shown in Supporting Information Table S3.

**TABLE 2:** Associations between anthropometric markers and incident type 2 diabetes by sex and women to men ratios of HRs on type 2 diabetes incidence

	Women				Men				Sex interaction	
	Total	Events	HR [95% CI]	*p* value	Total	Events	HR [95% CI]	*p* value	Ratio of HR^a^ (HR_men_/HR_women_)	*p* value
Height	88,557	2638	0.92 [0.89–0.96]	<0.001	72,570	3677	0.96 [0.93–0.99]	0.019	1.05 [1.00–1.11]	0.059
Weight			1.85 [1.79–1.91]	<0.001			1.77 [1.73–1.82]	<0.001	0.96 [0.92–0.99]	0.024
ABSI			1.51 [1.46–1.57]	<0.001			1.23 [1.18–1.27]	<0.001	0.81 [0.77–0.85]	<0.001
BMI			1.93 [1.88–1.99]	<0.001			1.85 [1.80–1.90]	<0.001	0.96 [0.92–0.99]	0.014
WC			1.69 [1.64–1.74]	<0.001			1.59 [1.55–1.63]	<0.001	0.94 [0.90–0.98]	0.002
HC			1.69 [1.64–1.74]	<0.001			1.59 [1.55–1.63]	<0.001	0.94 [0.90–0.98]	0.002
WHR			2.03 [1.96–2.10]	<0.001			1.46 [1.44–1.48]	<0.001	0.72 [0.69–0.75]	<0.001
WHtR			2.27 [2.19–2.35]	<0.001			1.96 [1.90–2.01]	<0.001	0.86 [0.83–0.90]	<0.001
VAI			1.46 [1.43–1.49]	<0.001			1.40 [1.37–1.43]	<0.001	0.96 [0.93–0.98]	0.001
HI			0.46 [0.45–0.48]	<0.001			0.51 [0.49–0.52]	<0.001	1.09 [1.04–1.15]	<0.001
ARI			1.70 [1.66–1.75]	<0.001			1.60 [1.57–1.64]	<0.001	0.94 [0.91–0.97]	<0.001

*Note*: Data are presented as hazard ratios (HR) with 95% CIs per 1‐SD increment in each adiposity marker.

Abbreviations: ABSI, A Body Shape Index; ARI, anthropometric risk index; HC, hip circumference; HI, hip index; VAI, visceral adiposity index; WC, waist circumference; WHR, waist to hip ratio; WHtR, waist to height ratio.

^a^ Hazard ratios above 1 suggest a higher risk in women compared with men, whereas hazard ratios below 1 suggest a higher risk in men compared with women. The model was adjusted for age, deprivation, smoking, alcohol, fruits and vegetables, red meat, processed meat, type of physical activity, and leisure screen time. All analyses were conducted using 2‐year landmark analyses and excluding participants with type 1, type 2, or unknown diabetes at baseline. SD for height 9.25, SD for weight 15.19, SD for ABSI 0.01, SD for BMI 4.46, SD for WC 12.83, SD for HC 8.62, SD for WHR 0.09, SD for WHtR 0.07, SD for VAI (women) 1.68, SD for VAI (men) 1.45, SD for HI (women) 0.01, SD for HI (men) 0.01, and SD for ARI 1.55.

**FIGURE 1:**
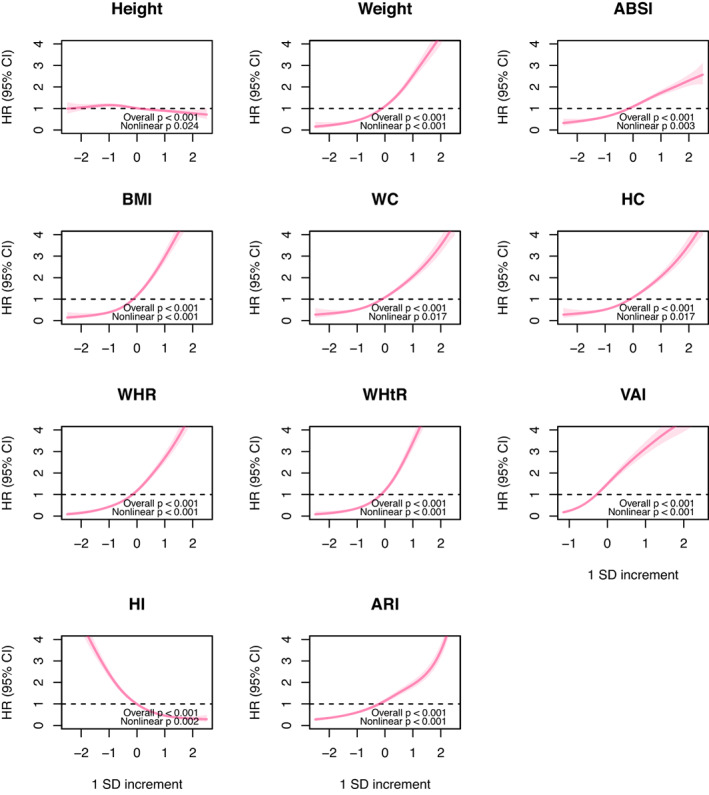
Dose–response associations between anthropometric markers and incident type 2 diabetes in women. Penalized splines were used to present the associations between anthropometric markers and incident type 2 diabetes. The anthropometric markers were sex‐standardized to a 1‐SD increment. Analyses were adjusted for sex, age, deprivation, smoking, alcohol, fruits and vegetables, red and processed meat, type of physical activity, and leisure screen time. All analyses were conducted using 2‐year landmark analyses and excluding participants with type 1, type 2, or unknown diabetes at baseline. ABSI, A Body Shape Index; ARI, anthropometric risk index; HI, hip index; HC, hip circumference; HR, hazard ratio; WHR, waist to hip ratio; WC, waist circumference; WHtR, waist to height ratio; VAI, visceral adiposity index [Color figure can be viewed at wileyonlinelibrary.com]

**FIGURE 2:**
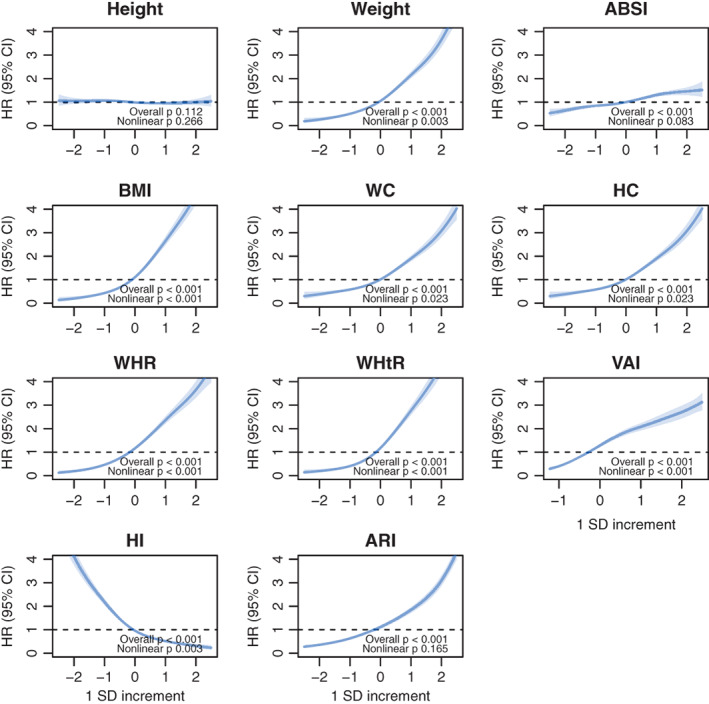
Dose–response associations between anthropometric markers and incident type 2 diabetes in men. Penalized splines were used to present the associations between anthropometric markers and incident type 2 diabetes. The anthropometric markers were sex‐standardized to a 1‐SD increment. Analyses were adjusted for sex, age, deprivation, smoking, alcohol, fruits and vegetables, red and processed meat, type of physical activity, and leisure screen time. All analyses were conducted using 2‐year landmark analyses and excluding participants with type 1, type 2, or unknown diabetes at baseline. ABSI, A Body Shape Index; ARI, anthropometric risk index; HI, hip index; HC, hip circumference; HR, hazard ratio; WHR, waist to hip ratio; WC, waist circumference; WHtR, waist to height ratio; VAI, visceral adiposity index [Color figure can be viewed at wileyonlinelibrary.com]

The dose–response associations between anthropometric markers and type 2 diabetes are shown in Figure 1 for women and Figure 2 for men. Most markers showed positive monotonic associations with type 2 diabetes, except height and HI, which were inversely associated with type 2 diabetes risk. The dose–response associations for all participants are shown in Supporting Information Figure S3.

The C indices are shown in Table 3. Among women, WHtR (0.80 [95% CI: 0.79–0.80]) and ARI (0.79 [95% CI: 0.78–0.79]) had a better predictive ability for incident type 2 diabetes compared with BMI (0.78 [95% CI: 0.77–0.78]). Similarly, WHtR (0.75 [95% CI: 0.74–0.75]) and ARI (0.74 [95% CI: 0.73–0.75]) were more predictive than BMI (0.74 [95% CI: 0.73–0.74]) among men (Table 3), as well as among all participants (Supporting Information Table S4).

**TABLE 3:** C index of comparison of BMI with anthropometric markers by sex

	Adiposity markers [95% CI]	BMI [95% CI]	ΔC [95% CI]	*p* value
Women				
Height	0.67 [0.66 to 0.68]	0.78 [0.77 to 0.78]	0.11 [0.10 to 0.12]	<0.001
Weight	0.76 [0.75 to 0.77]		0.02 [0.02 to 0.02]	<0.001
ABSI	0.71 [0.70 to 0.72]		0.07 [0.06 to 0.08]	<0.001
WC	0.74 [0.73 to 0.74]		0.04 [0.04 to 0.04]	<0.001
HC	0.74 [0.73 to 0.74]		0.04 [0.04 to 0.04]	<0.001
WHR	0.77 [0.76 to 0.77]		0.01 [0.00 to 0.02]	0.0132
WHtR	0.80 [0.79 to 0.80]		−0.02 [−0.02 to −0.02]	<0.001
VAI	0.75 [0.74 to 0.76]		0.03 [0.02 to 0.03]	<0.001
HI	0.76 [0.75 to 0.77]		0.02 [0.02 to 0.02]	<0.001
ARI	0.79 [0.78 to 0.79]		−0.01 [−0.01 to −0.01]	<0.001
Men				
Height	0.63 [0.62 to 0.64]	0.74 [0.73 to 0.74]	0.11 [0.10 to 0.12]	<0.001
Weight	0.71 [0.70 to 0.72]		0.03 [0.02 to 0.03]	<0.001
ABSI	0.64 [0.63 to 0.65]		0.10 [0.09 to 0.11]	<0.001
WC	0.69 [0.68 to 0.70]		0.05 [0.04 to 0.05]	<0.001
HC	0.69 [0.68 to 0.70]		0.05 [0.04 to 0.05]	<0.001
WHR	0.71 [0.70 to 0.72]		0.03 [0.02 to 0.03]	<0.001
WHtR	0.75 [0.74 to 0.75]		−0.01 [−0.01 to 0.00]	0.0001
VAI	0.69 [0.68 to 0.70]		0.05 [0.04 to 0.06]	<0.001
HI	0.71 [0.71 to 0.72]		0.03 [0.02 to 0.03]	<0.001
ARI	0.743 [0.735 to 0.751]		−0.004 [−0.006 to −0.001]	0.0116

*Note*: ΔC [95% CI], differences between C indices with the BMI model and their 95% CIs; *p* value for ΔC. The analysis was adjusted for age, systolic blood pressure, and family history of diabetes. All analyses excluded participants with type 1, type 2, or unknown diabetes at baseline.

Abbreviations: ABSI, A Body Shape Index; ARI, anthropometric risk index; WHR, waist to hip ratio; HC, hip circumference; HI, hip index; VAI, visceral adiposity index; WC, waist circumference; WHtR, waist to height ratio.

## DISCUSSION

The main finding of this study was that most anthropometric markers were associated with type 2 diabetes, regardless of sociodemographics, diet, and physical activity. Except for height and HI, higher values of all markers were associated with a higher risk of type 2 diabetes monotonically. These findings are the first study reporting the prospective associations of more complex measures of ABSI, ARI, HI, and VAI with incident type 2 diabetes among White Europeans [8].

Our findings corroborated the previous findings in the recent systematic review of prospective and retrospective cohort studies that BMI (relative risk [RR]: 1.72), WC (RR 1.61), WHR (RR 1.63), WHtR (RR 1.73), VAI (RR 1.42), ABSI (RR 1.09) and HC (RR 1.11) were associated with a higher incidence of type 2 diabetes [12]. However, this systematic review included some smaller studies with strong selection bias; therefore, the findings might not be as robust as in our large general population study. We provided new findings that a 1‐SD increment in ARI was associated with a higher risk of type 2 diabetes. Likewise, an ARI study based on combining height, weight, and waist and hip measurements reported that height (RR 0.96) and HI (HR ~0.92–0.99) were associated with a lower risk of mortality [8]. Our study extends the findings to show the associations between height and HI with a lower type 2 diabetes risk, consistent with previous studies [24, 25]. Therefore, this would imply that being shorter (among females) and having smaller hips were associated with a higher risk of type 2 diabetes. However, our findings did not agree with a previous study about the association between ABSI and the incidence of type 2 diabetes. For example, there was no association between ABSI and incident type 2 diabetes for both women and men, but this study was conducted in older people [26].

Moreover, the aforementioned systematic review and meta‐analysis did not examine the C index to evaluate the predictive performance [12]. Our findings used the C index to explore the predictive ability of adiposity markers and found that WHtR and ARI were better predictors, whereas the rest of the markers were poorer predictors relative to BMI. It appears that WHtR is the strongest candidate as an adiposity marker to predict type 2 diabetes in all participants. Given that the UK Biobank is not representative of the general population, the findings should be externally validated. Although the changes in the C indices in our study may appear modest, when applied to the population they could mean substantial prevention [27, 28]. Notably, the improvement of the C index in our study (ΔC_WHtR_ 0.01) was larger than when total and high‐density lipoprotein cholesterol was added to cardiovascular disease prediction (ΔC = 0.004) [29], both of which are staples of risk prediction.

Interestingly, ABSI was previously found to be a good predictor among Chinese and Japanese populations [30, 31]. However, the results are still inconsistent among other studies because some found that the predictive ability of ABSI for type 2 diabetes was not better than WC, BMI [32], and VAI [33]. Nonetheless, our findings are consistent: ABSI was also not better than other anthropometric markers among White Europeans in the UK Biobank.

Our findings have important public health relevance showing that relatively simple measurements, such as BMI or WHtR, have similar associations with more complex markers, such as VAI or ARI. This might suggest that the relatively simple adiposity marker is sufficient in clinical settings to reflect type 2 diabetes risk [34]. The current findings suggest that for type 2 diabetes risk prediction and stratification, WHtR is the strongest candidate for the White European population. A further multinational comparison should investigate whether this differs by ethnic group. In addition, to translate these findings into clinical practice, future studies should explore the optimal cutoff values of these anthropometric measures, particularly WHtR, for type 2 diabetes screening.

The large sample size of this study allowed us to explore the associations between anthropometric markers and incident type 2 diabetes and risk prediction. Anthropometric markers were measured by trained staff using standardized protocols, which could imply that the values were valid. Our findings clearly show that anthropometric markers were associated with a higher risk of type 2 diabetes in White people. Our findings addressed the limitations of previous evidence about the methodology and report of the development of the risk prediction models for type 2 diabetes, specifically on the number of participants, how continuous variables were treated, and reporting of missing data [35].

However, the present study is not exempt from limitations. First and foremost, we were unable to include other ethnic groups in this study due to their small proportions compared with the White population; thus, the findings cannot be extrapolated to other ethnic populations. Secondly, the UK Biobank is not representative of the general population of the UK in terms of sociodemographic, physical, lifestyle, and health‐related characteristics of the general population. Although the evidence showed healthy volunteer selection bias, exposure–disease risk estimates should be generalized to the broader population and lifestyle‐related factors [36]. Thirdly, our study used ICD‐10 to define our outcome. Therefore, any study that uses our findings should be aware of the definitional error according to ICD‐10. For example, previous evidence has shown that ICD‐9 and ICD‐10 do not always align and that the transition to ICD‐10 could lead to a discontinuity over a period of time [37]. Lastly, we could not rule out unobserved, unmeasured confounding as with any observational studies.

In conclusion, even though most anthropometric markers were associated with a higher risk of type 2 diabetes, the magnitude of the associations differed. WHtR had the strongest associations and predictive ability for type 2 diabetes and thus could be a more suitable marker for clinical use.

## AUTHOR CONTRIBUTIONS

Jirapitcha Boonpor, Solange Parra‐Soto, Stuart R. Gray, Carlos Celis‐Morales, and Frederick K. Ho contributed to the study conception and design. Jirapitcha Boonpor, Solange Parra‐Soto, Atefeh Talebi, Carlos Celis‐Morales, and Frederick K. Ho performed the statistical analyses. Stuart R. Gray, Carlos Celis‐Morales, and Frederick K. Ho advised on all statistical aspects. Jirapitcha Boonpor, Solange Parra‐Soto, Atefeh Talebi, Stuart R. Gray, Carlos Celis‐Morales, and Frederick K. Ho interpreted the data. Jirapitcha Boonpor, Solange Parra‐Soto, Stuart R. Gray, Carlos Celis‐Morales, and Frederick K. Ho drafted the manuscript. All authors (Jirapitcha Boonpor, Solange Parra‐Soto, Atefeh Talebi, Ziyi Zhou, Fernanda Carrasco‐Marin, Fanny Petermann‐Rocha, Paul Welsh, Jill P. Pell, Naveed Sattar, Jason M. R. Gill, Stuart R. Gray, Carlos Celis‐Morales, and Frederick K. Ho) contributed to the acquisition of data. All authors critically revised the manuscript. All authors read and approved the final article.

## FUNDING INFORMATION

The UK Biobank was established by the Wellcome Trust; Medical Research Council; Department of Health, Scottish Government; and the Northwest Regional Development Agency. It has also received funding from the Welsh Assembly Government and the British Heart Foundation. Jirapitcha Boonpor receives financial support from the Royal Thai Government Scholarship for her PhD. Solange Parra‐Soto receives financial support from the Chilean Government PhD scholarship program for their PhD. The funders had no role in the study design; in the collection, analysis, and interpretation of data; in the writing of the report; and in the decision to submit the paper for publication.

## CONFLICT OF INTEREST STATEMENT

The authors declared no conflict of interest.

## Supporting information


**Data S1.** Supporting information.

## Data Availability

Data can be requested from the UK Biobank (https://www.ukbiobank.ac.uk/).
