# Supplementary material for: Associations and predictive performance of 11 anthropometric measures with incident type 2 diabetes: A prospective cohort study from the UK Biobank
Source: Obesity (Silver Spring). 2023 Sep 19;31(10):2648–57. doi: 10.1002/oby.23849 (PMC10947384; doi:10.1002/oby.23849)
Supplement: Supplementary file 1 — Data S1. Supporting information. [file OBY-31-2648-s001.docx]

**Supplementary Table S1**. Anthropometric adiposity-related markers formulas

| **Markers** | **Formulas** |
| --- | --- |
| A Body Shape Index (ABSI)(1) | Waist circumference / (Body Mass Index^2/3^ × Height^1/2^) |
| Hip Index_women_ (HI)(2) | Hip circumference × Weight ^-0.482^× Height^0.310^ |
| Hip Index_men_ (HI)(2) | Hip circumference × Weight ^-2/5^ × Height^1/5^ |
| Visceral Adiposity Index_women_ (VAI)(3) | (Waist circumference / 36.58 + (1.89 × Body Mass Index)) × (Triglyceride / 0.81) × (1.52 / High-density lipoprotein cholesterol) |
| Visceral Adiposity Index_men_ (VAI)(3) | (Waist circumference / 39.68 + (1.88 × Body Mass Index)) × (Triglyceride / 1.03) × (1.31 / High-density lipoprotein cholesterol) |

**Supplementary Table S2**. Characteristics by sex

| Characteristics | Women  88,557 (55.0%) | Men  72,570 (45.0%) |
| --- | --- | --- |
| **Age** (year), mean ± SD | 56.5 ± 7.9 | 56.7 ± 8.1 |
| **Townsend deprivation index**, n (%) |  |  |
| Lower deprivation | 31428 (35.5) | 26208 (36.1) |
| Middle deprivation | 31357 (35.4) | 25285 (34.8) |
| Higher deprivation | 25772 (29.1) | 21077 (29.1) |
| **Smoking**, n (%) |  |  |
| Never | 52969 (59.8) | 36601 (50.5) |
| Previous | 28344 (32.0) | 27890 (38.4) |
| Current | 7244 (8.2) | 8079 (11.1) |
| **Alcohol intake**, n (%) |  |  |
| Daily or almost daily | 14903 (16.8) | 18962 (26.1) |
| 3-4 times a week | 19506 (22.1) | 20484 (28.2) |
| Once or twice a week | 24215 (27.3) | 19412 (26.8) |
| 1-3 times a month | 11803 (13.3) | 6190 (8.5) |
| Special occasions only | 11661 (13.2) | 4281 (5.9) |
| Never | 6469 (7.3) | 3241 (4.5) |
| **Body mass index** (kg/m^2^), mean ± SD | 26.8 ± 4.8 | 27.6 ± 3.9 |
| **Height** (cm), mean ± SD | 162.6 ± 6.2 | 175.9 ± 6.8 |
| **WC** (cm), mean ± SD | 83.6 ± 11.7 | 96.1 ± 10.6 |
| **Weight** (kg), mean ± SD | 70.7 ± 13.2 | 85.4 ± 13.5 |
| **ABSI**, mean ± SD | 0.07 ± 0.005 | 0.08 ± 0.004 |
| **HC** (cm), mean ± SD | 102.8 ± 9.7 | 103.1 ± 7.0 |
| **WHR**, mean ± SD | 0.8 ± 0.07 | 0.9 ± 0.06 |
| **WHtR**, mean ± SD | 0.5 ± 0.07 | 0.6 ± 0.06 |
| **ARI**, mean ± SD | -0.2 ± 1.58 | -0.1 ± 1.52 |
| **HI**, mean ± SD | 0.07 ± 0.008 | 0.07 ± 0.006 |
| **VAI**, mean ± SD | 1.9 ± 1.5 | 2.2 ± 1.7 |
| **Fruits and vegetables** (portion/day), mean ± SD | 4.4 ± 2.2 | 3.8 ± 2.3 |
| **Red meat** (portion/week), mean ± SD | 2.0 ± 1.3 | 2.3 ± 1.4 |
| **Processed meat** (portion/week), mean ± SD | 1.6 ± 1.0 | 2.2 ± 1.0 |
| **Leisure screen time** (hour/day), mean ± SD | 4.6 ± 1.9 | 5.4 ± 2.3 |
| **Sleeping time**, n (%) |  |  |
| <7 hrs a day | 66972 (75.6) | 54448 (75.0) |
| 7 – 8 hrs a day | 20207 (22.8) | 17184 (23.7) |
| >9 hrs a day | 1378 (1.6) | 938 (1.3) |
| **Type of PA**, n (%) |  |  |
| Walking for pleasure | 70715 (79.9) | 53723 (74.0) |
| Other exercises | 10263 (11.6) | 10034 (13.8) |
| Strenuous sports | 298 (0.3) | 998 (1.4) |
| Light DIY | 5917 (6.7) | 5017 (6.9) |
| Heavy DIY | 1364 (1.5) | 2798 (3.9) |

Data are presented as mean ± SD for continuous variables and as n (%) for categorical variables.

ABSI, A Body Shape Index; ARI, anthropometric risk index; DIY, do-it-yourself; HC, hip circumference; HI, hip index; PA, physical activity; WC, waist circumference; WHR, waist-to-hip ratio; WHtR, waist-to-height ratio; VAI, visceral adiposity index

**Supplementary Table S3**. Association between anthropometric markers and type 2 diabetes incident in white Europeans.

| Marker | Total | Event | Model 1 |  | Model 2 |  |
| --- | --- | --- | --- | --- | --- | --- |
|  | 161,127 | 6,315 | HR (95% CI) | p-value | HR (95% CI) | p-value |
| Height |  |  | 0.92 (0.89; 0.94) | <0.001 | 0.94 (0.92; 0.97) | <0.001 |
| Weight |  |  | 1.89 (1.85; 1.92) | <0.001 | 1.81 (1.77; 1.84) | <0.001 |
| ABSI |  |  | 1.39 (1.35; 1.42) | <0.001 | 1.35 (1.31; 1.38) | <0.001 |
| BMI |  |  | 1.96 (1.93; 2.00) | <0.001 | 1.88 (1.85; 1.92) | <0.001 |
| WC |  |  | 1.71 (1.68; 1.74) | <0.001 | 1.63 (1.60; 1.66) | <0.001 |
| HC |  |  | 1.71 (1.68; 1.74) | <0.001 | 1.63 (1.60; 1.66) | <0.001 |
| WHR |  |  | 1.50 (1.49; 1.52) | <0.001 | 1.51 (1.5; 1.53) | <0.001 |
| WHtR |  |  | 2.17 (2.13; 2.22) | <0.001 | 2.08 (2.03; 2.12) | <0.001 |
| VAI |  |  | 1.49 (1.47; 1.50) | <0.001 | 1.43 (1.41; 1.45) | <0.001 |
| HI |  |  | 0.46 (0.45; 0.47) | <0.001 | 0.49 (0.48; 0.50) | <0.001 |
| ARI |  |  | 1.70 (1.67; 1.72) | <0.001 | 1.64 (1.62; 1.67) | <0.001 |

Data are presented as hazard ratios (HRs) with their confidence intervals (95% CIs) per 1 standard deviation increment in each adiposity marker. Model 1 was adjusted for sex, age, and deprivation. Model 2 was included for Model 1 plus smoking, alcohol, fruit & vegetables, red meat, processed meat, type of physical activity and leisure screen time. All analyses were conducted using 2-year landmark analyses and excluding participants with type 1, type 2 diabetes or unknown diabetes at baseline. SD for height; 9.25, weight; 15.15, ABSI; 0.01, BMI; 4.45, WC; 12.8, HC; 8.6, WHR; 0.09, WHtR; 0.07, VAI (women); 1.67, VAI (men); 1.45, HI (women); 0.01, HI (men); 0.01 and ARI; 1.55.

ABSI, A Body Shape Index; ARI, anthropometric risk index; BMI, body mass index; HC, hip circumference; HI, hip index; WC, waist circumference; WHR, waist-to-hip ratio; WHtR, waist-to-height ratio; VAI, visceral adiposity index

**Supplementary Table S4**. C-index of comparison of BMI with anthropometric markers for all participants

| Marker | Adiposity Markers (95% CI) | BMI (95% CI) | ΔC (95% CI) | p-value |
| --- | --- | --- | --- | --- |
| Height | 0.66 [0.66; 0.67] | 0.76 [0.76; 0.77] | 0.10 [0.10; 0.11] | <0.001 |
| Weight | 0.74 [0.74; 0.75] |  | 0.02 [0.02; 0.02] | <0.001 |
| ABSI | 0.69 [0.68; 0.69] |  | 0.08 [0.07; 0.08] | <0.001 |
| WC | 0.72 [0.72; 0.73] |  | 0.04 [0.04; 0.04] | <0.001 |
| HC | 0.72 [0.72; 0.73] |  | 0.04 [0.04; 0.04] | <0.001 |
| WHR | 0.74 [0.74; 0.75] |  | 0.02 [0.02; 0.03] | <0.001 |
| WHtR | 0.78 [0.77; 0.78] |  | -0.01 [-0.02; -0.01] | <0.001 |
| VAI | 0.73 [0.72; 0.74] |  | 0.03 [0.03; 0.04] | <0.001 |
| HI | 0.74 [0.74; 0.75] |  | 0.02 [0.02; 0.02] | <0.001 |
| ARI | 0.77 [0.77; 0.78] |  | -0.01 [-0.01; -0.01] | <0.001 |

ΔC (95% CI): Difference between C-indices with the BMI model and their 95% confidence interval, P: p-value for ΔC.

The analysis was adjusted for age, sex, systolic blood pressure and family history of diabetes. All analyses excluded participants with type 1, type 2 diabetes or unknown diabetes at baseline.

ABSI, A Body Shape Index; ARI, anthropometric risk index; BMI, body mass index; HC, hip circumference; HI, hip index; WC, waist circumference; WHR, waist-to-hip ratio; WHtR, waist-to-height ratio; VAI, visceral adiposity index

UK Biobank participants during 2006 – 2010

N=502,411

**Exclusion**

No linked primary care records: 273,962

Available primary care data

N=228,449

**Exclusion**

People with non-white background: 11667

White participants

N=216,782

**Exclusions**

Type 2 diabetes at baseline: 10994

Undiagnosed diabetes: 1357

Missing covariates: 16086

Missing exposures: 26593

Incident type 2 diabetes occurred in the first 2 years of follow-up: 625

UK Biobank participants were included in the study

N=161,127

**Supplementary Figure S1**. Flow chart of participants

**
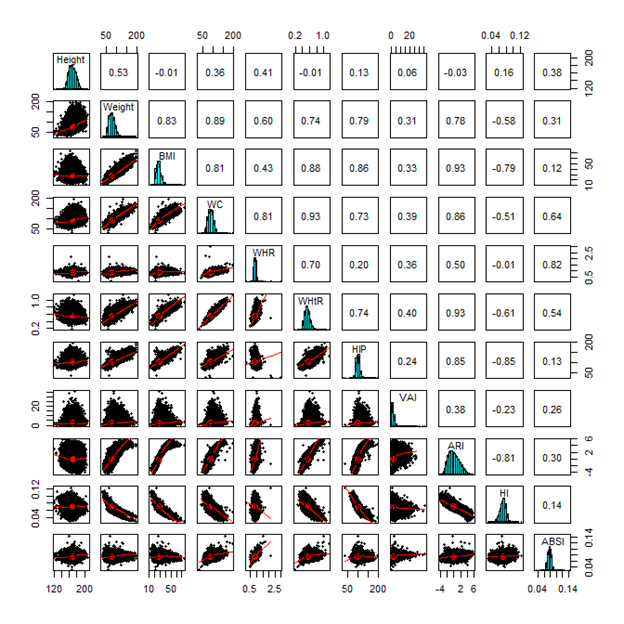
**

**Supplementary Figure S2**. Pearson correlation coefficients between adiposity markers

All analyses were conducted using 2-year landmark analyses, excluding participants with type 1, type 2 diabetes or unknown diabetes.

ABSI, A Body Shape Index; ARI, anthropometric risk index; BMI, body mass index; HC, hip circumference; HI, hip index; WC, waist circumference; WHR, waist-to-hip ratio; WHtR, waist-to-height ratio; VAI, visceral adiposity index

_
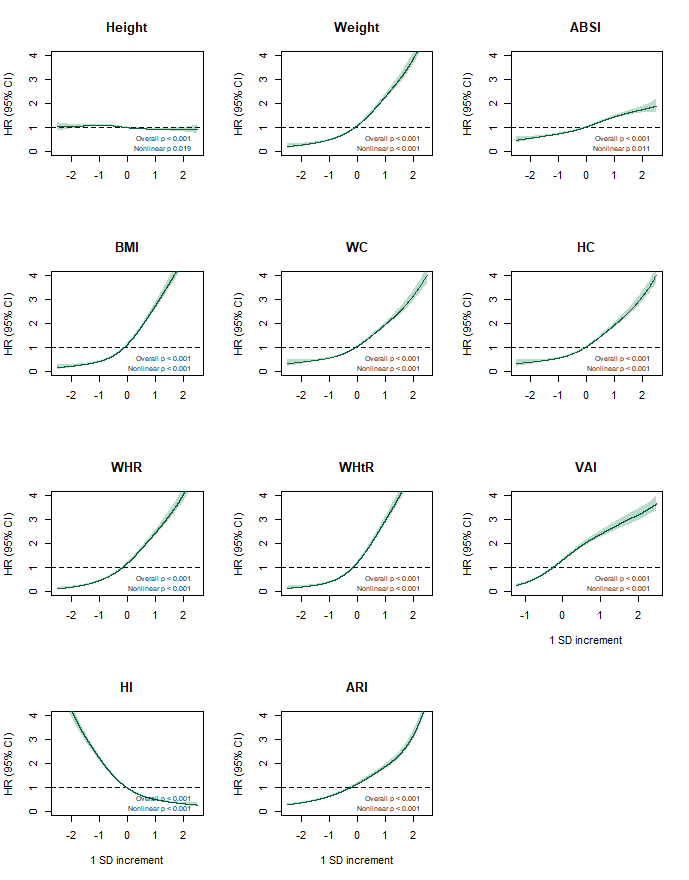
_

**Supplementary Figure S3**. Association between anthropometric markers and incident type 2 diabetes among all participants.

Penalised splines were used to present the association between anthropometric markers and incident type 2 diabetes. The anthropometric markers were sex-standardised to 1-SD increment. Analyses were adjusted for sex, age, deprivation, smoking, alcohol, fruit & vegetables, red & processed meat, type of physical activity and leisure screen time. All analyses were conducted using 2-year landmark analyses and excluding participants with type 1, type 2 diabetes or unknown diabetes at baseline.

ABSI, A Body Shape Index; ARI, anthropometric risk index; BMI, body mass index; HC, hip circumference; HI, hip index; WC, waist circumference; WHR, waist-to-hip ratio; WHtR, waist-to-height ratio; VAI, visceral adiposity index

**References**

1. Krakauer NY, Krakauer JC. A new body shape index predicts mortality hazard independently of body mass index. *PLoS One* 2012;**7:** e39504.

2. Christakoudi S, Tsilidis KK, Evangelou E, Riboli E. Association of body-shape phenotypes with imaging measures of body composition in the UK Biobank cohort: relevance to colon cancer risk. *BMC Cancer* 2021;**21:** 1106.

3. Amato MC, Giordano C, Galia M, Criscimanna A, Vitabile S, Midiri M*, et al.* Visceral Adiposity Index: a reliable indicator of visceral fat function associated with cardiometabolic risk. *Diabetes Care* 2010;**33:** 920-922.
